# Genome-Wide Association Study of Glucosinolate Metabolites (mGWAS) in *Brassica napus* L.

**DOI:** 10.3390/plants12030639

**Published:** 2023-02-01

**Authors:** Yunshan Tang, Guorui Zhang, Xinyue Jiang, Shulin Shen, Mingwei Guan, Yuhan Tang, Fujun Sun, Ran Hu, Si Chen, Huiyan Zhao, Jiana Li, Kun Lu, Nengwen Yin, Cunmin Qu

**Affiliations:** 1 Chongqing Engineering Research Center for Rapeseed, College of Agronomy and Biotechnology, Southwest University, Chongqing 400716, China; 2 Academy of Agricultural Sciences, Southwest University, Chongqing 400715, China; 3 Affiliation Engineering Research Center of South Upland Agriculture, Ministry of Education, Chongqing 400715, China

**Keywords:** *Brassica napus*, glucosinalates, mGWAS, candidate genes

## Abstract

Glucosinolates (GSLs) are secondary plant metabolites that are enriched in rapeseed and related *Brassica* species, and they play important roles in defense due to their anti-nutritive and toxic properties. Here, we conducted a genome-wide association study of six glucosinolate metabolites (mGWAS) in rapeseed, including three aliphatic glucosinolates (m145 gluconapin, m150 glucobrassicanapin and m151 progoitrin), one aromatic glucosinolate (m157 gluconasturtiin) and two indole glucosinolates (m165 indolylmethyl glucosinolate and m172 4-hydroxyglucobrassicin), respectively. We identified 113 candidate intervals significantly associated with these six glucosinolate metabolites. In the genomic regions linked to the mGWAS peaks, 187 candidate genes involved in glucosinolate biosynthesis (e.g., *BnaMAM1*, *BnaGGP1*, *BnaSUR1* and *BnaMYB51*) and novel genes (e.g., *BnaMYB44*, *BnaERF025*, *BnaE2FC*, *BnaNAC102* and *BnaDREB1D*) were predicted based on the mGWAS, combined with analysis of differentially expressed genes. Our results provide insight into the genetic basis of glucosinolate biosynthesis in rapeseed and should facilitate marker-based breeding for improved seed quality in *Brassica* species.

## 1. Introduction

Glucosinolates (GSLs) are secondary metabolites comprising sulfur and nitrogen that are specially produced in *Brassica* species, providing these plants with their pungent odor [1,2,3]. GSLs also play important roles in plant defense against pests and in human health [4,5]. However, high levels of GSLs affect the quality of seed oil and the nutritional value of seed meal from rapeseed. In addition, some hydrolysates produced from GSLs, such as oxazolidin-2-thione, have toxic effects on human and animal health [6,7,8,9,10]. Thus, further reducing glucosinolate levels in seed meal is an important goal in rapeseed breeding.

Generally, GSLs share the same basic structure, including *β*-D-thiosaccharide and (Z)-*n*-hydroxamic sulfate, but have variable R-side chain groups due to the precursor amino acids [11]. The lengths and modifications of these variable R-side chain groups determine the chemical properties of each GSL. Correspondingly, GSLs can be divided into three types based on the source of precursor amino acids, for example, aliphatic GSLs derived from alanine (Ala), leucine (Leu), isoleucine (Ile), methionine (Met) and valine (Val); indole GSLs derived from tryptophan (Trp); and aromatic GSLs derived from phenylalanine (Phe) and tyrosine (Tyr) [12,13,14]. To date, over 200 GSLs have been discovered [15,16], which were usually synthesized through three steps. The first step is the lengthening of the progenitor amino acid side chain. This mainly occurs during the biosynthesis of aliphatic and aromatic GSLs, in which amino acids form 2-oxic acids via the action of branched-chain amino acid transaminase (BCAT) and then undergo elongation by enzymes, such as methylthinomalate synthase (MAM), isopropyl malate isomerase (IPMI) and isopropyl malate dehydrogenase (IMD). The extended 2-oxic acid molecule is converted into the corresponding amino acid by BCAT [5,17,18,19]. The second step forms the glucosinolate core structure. The extended amino acid is converted via the action of cytochrome P450 79s (CYP79s) to aldoxime, which is oxidized into the activated forms by CYP83s. These activated forms could bind to glutathione that is converted to first thiohydroxamic acid by glutathione S-transferases (GSTFs), *γ*-glutamyl peptide (GGP1) and C-S lyase (Super root 1 (SUR1)), and then to the GSL core structure by glucosyltransferases (UGT74s) and sulfotransferases (SOTs) [5,20,21,22,23,24]. The last step involves side chain modification of the core structure, primarily through hydroxylation, sulfation, glycosylation, desaturation and methylation of R groups [25]. The core structures of aliphatic GSLs are oxidized and hydroxylated by a series of enzymes, including flavin monooxygenase (FMOGS-OX) and alkenyl hydroxalkyl-producing proteins (AOPs) for aliphatic GSLs, 2-oxoglutarate-dependent dioxygenase (GS-OH) for aromatic GSLs and CYP81Fs and indole glucosinolate *O*-methyltransferases (IGMTs) for indole GSLs [26,27,28].

In *Arabidopsis*, the glucosinolate biosynthesis pathway is one of the best characterized specialized metabolite pathways [12]. Progress has also been made in mining many genes associated with the GSL pathway. Many catalytic enzymes that function in the biosynthesis of different GSLs have been identified, such as CYP79A2, CYP83B1, SUR1, UGT74B1, sulfotransferase (ST5A) and so on [29,30,31,32,33,34]. In addition, some MYB transcription factors have also been shown to regulate the expression of genes encoding enzymes in the GSL biosynthesis pathway. For example, AtMYB51 activates the transcription of GSL-biosynthesis-related genes (*AtTSB1*, *AtCYP79B2*, *AtCYP79B3*, *AtCYP83B1* and *AtST5a* and so on), leading to the accumulation of GSLs [35]. The aliphatic GSL pathway is regulated by MYB28 and MYB29 [36,37], while indole GSL biosynthesis is jointly regulated by MYB34, MYB51 and MYB122 [38,39]. Several MYC transcription factors also function in GSL biosynthesis by regulating the jasmonic acid metabolism pathway [40]. Furthermore, MYC2, MYC3 and MYC4 interact with multiple MYB transcription factors (e.g., MYB28, MYB29, MYB34, MYB51 and MYB76), thereby contributing to GSL biosynthesis [40,41]. A recent study showed that WRKY33 not only directly targets the promoters of the gene *MYB51* and the GSL biosynthesis gene *CYP83B1* to regulate the de novo biosynthesis of indole GSLs, but also regulates the expression of genes involved in side chain modification (*CYP81F2*, *IGMT1* and *IGMT2*), thereby increasing the biosynthesis of indole GSLs [42].

Rapeseed (*Brassica napus* L.) is used worldwide as an oil crop and source of edible vegetable oil and feed meal. Therefore, breeding rapeseed varieties with low GSL levels in seeds is an important breeding goal. To date, many rapeseed resources with low seed GSL contents have been developed in polyploid rapeseed [43,44,45]. However, due to the complexity of the rapeseed genome, identifying the GSL biosynthesis pathway in rapeseed and the mechanism leading to low GSL content in *B*. *napus* seeds has been challenging. In this study, we performed a genome-wide association study for six glucosinolate metabolites in 143 rapeseed accessions using 239,945 SNP markers obtained by resequencing [46,47,48]. Our goals were to identify candidate genes to enrich the metabolic regulatory network of the GSL biosynthesis pathway, and to lay a foundation for the breeding of *B. napus* with improved quality.

## 2. Results

### 2.1. Identification and Statistical Analysis of Glucosinolate Metabolites in B. napus

**Figure 1:**
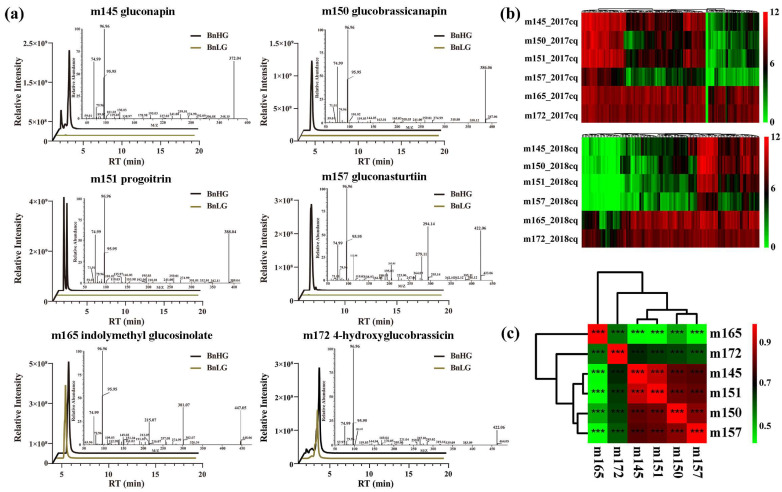
Characterization of six glucosinolate metabolites in *B. napus* seeds. (**a**) Ion peaks of six GSLs in seeds at 35 DAP, including MS and MS/MS spectra. BnHG represents the rapeseed accession Zhongyou 821 with high total GSL contents, and BnLG represents rapeseed accession Zhongshuang 11 with low total GSL contents. (**b**) Heatmap showing the variable accumulation of six glucosinolate metabolites in the population at 35 DAP. (**c**) Correlation analysis among the levels of the six glucosinolate metabolites. The asterisks indicate significant difference (***, *p* < 0.001).

**Figure 2:**
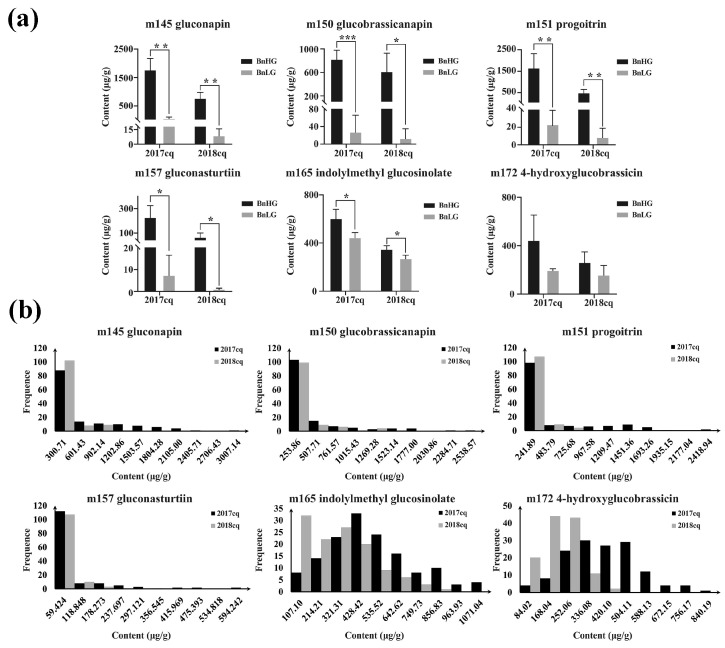
Variation in the contents of six glucosinolate metabolites in the rapeseed panel in different years (2017cq and 2018cq). (**a**) Differences in the contents of six glucosinolate metabolites in BnHG and BnLG. Asterisks indicate significant differences (*, *p* < 0.05; **, *p* < 0.01; and ***, *p* < 0.001). (**b**) Frequency distributions of the six glucosinolate metabolites for 143 rapeseed accessions in the two years (2017cq and 2018cq).

Based on ultrahigh-performance liquid chromatography-heated electrospray ionization-tandem mass spectrometry (UPLC-HESI-MS/MS) analysis, we obtained six glucosinolate metabolites with high content in rapeseed at 35 days after flowering (DAF), including three aliphatic GSLs (m145 gluconapin, m150 glucobrassicanapin and m151 progoitrin), one aromatic GSL (m157 gluconasturtiin) and two indole GSLs (m165 indolylmethyl-glucosinolate and m172 4-hydroxyglucobrassicin) (Figure 1a,b). Correlation analysis showed that the levels of three aliphatic GSLs (m145, m150 and m151) and aromatic GSL (m157) were positively correlated (|r| ≥ 0.8, *p* < 0.001), whereas the level of m172 showed a higher correlation with those of three aliphatic GSLs (|r| ≥ 0.6, *p* < 0.001) than with that of m165 (Figure 1c, Table 1 and Table S6 and Figure S4), indicating the consistency of these traits across various environments. Subsequently, we calculated the range, average, standard deviation, coefficient of variation, diversity index and heritability of the levels of the six glucosinolate metabolites (Table 1). The coefficient of variation among these metabolites ranged from 0.42 to 1.97, with an average of 1.31, and varied greatly under different years. The coefficient of variation of six glucosinolate metabolites in high glucosinolate content rapeseed was generally lower than those in low and medium glucosinolate content rapeseed (Table S7). Furthermore, genotype (G) × environment (E) interaction was also highly significant (Table 1), indicating that the accumulation levels in seeds are easily affected by environmental conditions. In addition, the order of the metabolites based on diversity index was m165 > m172 > m145 > m151 > m150 > m157, while the order based on heritability was m150 > m165 > m145 > m151 > m157 > m172 (Table 1). Therefore, all six glucosinolate metabolites not only exhibited obvious differences between BnHG and BnLG (Figure 2a), but also showed continuous variation in 2017cq and 2018cq (Figure 2b).

### 2.2. Genome-Wide Association Study of Glucosinolate Metabolites (mGWAS)

**Figure 3:**
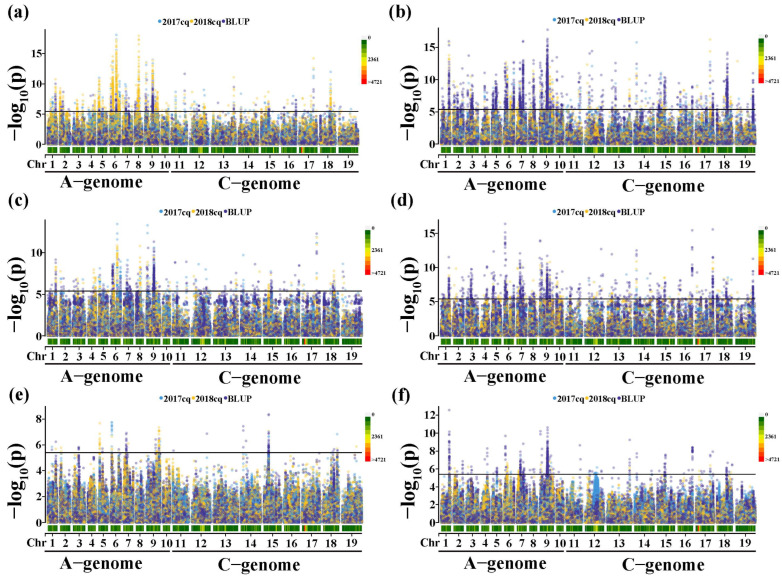
Manhattan plots of association analysis for six glucosinolate metabolites. (**a**) Manhattan plot for m145 in 2017cq, 2018cq and BLUP values. (**b**) Manhattan plot for m150 in 2017cq, 2018cq and BLUP values. (**c**) Manhattan plot for m151 in 2017cq, 2018cq and BLUP values. (**d**) Manhattan plot for m157 in 2017cq, 2018cq and BLUP values. (**e**) Manhattan plot for m165 in 2017cq, 2018cq and BLUP values. (**f**) Manhattan plot for m172 in 2017cq, 2018cq and BLUP values. Different-colored spots represent different environments. The horizontal lines indicate the Bonferroni-adjusted significance threshold (4.17 × 10^−6^).

To avoid identifying the false-positive associations in mGWAS, we selected six models to identify significant associations between phenotypes and genotypes. Based on the QQ plots of the six models (Figure S1), we performed GLM with Q model and MLM with Q+K model for the GWAS to mine more candidate genes of GSL metabolites, respectively. Herein, we used 239,945 high-quality SNPs (minor allele frequency (MAF) > 0.05 and the call frequencies <0.8) for the association analysis. The association signals were determined using a *p*-value < 4.17 × 10^−6^. The results of mGWAS for the six glucosinolate metabolites in two years (2017cq and 2018cq) and BLUP (best linear unbiased prediction) values (Figure 3, and Tables S1–S3) are summarized below. 

For the aliphatic GSL m145, we identified 1597, 2543 and 681 significantly associated SNPs based on 2017cq, 2018cqs and BLUP values, respectively, 389 SNPs of which were repeatedly detected in this study (Figure 3a, Figure S2a and Table S1). These significant SNPs primarily covered 79 candidate intervals located across the entire *B. napus* genome (Table S3). Importantly, two significant regions with high SNP densities were located on chromosomes A09 (17.88~22.59 Mb) and A06 (10.98~17.71 Mb) at the intervals designated qGSL-A09-5 and qGSL-A06-3, respectively. We found that the most significant SNPs (S6_16080408 and S9_18653335) could explain 49.67% and 43.75% of phenotypic variance, respectively. Therefore, we used these two intervals to predict the candidate genes to control the accumulation of the aliphatic GSL m145 in seeds (Table S3). In addition, 49 candidate regions were only associated with m145 in one or any two years and BLUP values. We believe that these remaining candidate regions represent minor-effect intervals influencing the accumulation of m145 (Table S3). These findings seem to suggest that m145 accumulation is strongly affected by environmental factors.

For the aliphatic glucosinolate, we identified m150, 2080, 2620 and 3444 significantly associated SNPs based on 2017cq, 2018cq and BLUP values, respectively, and 1431 SNPs were repeatedly detected in two years and BLUP values (Figure 3b and Figure S2b and Table S1). These significant SNPs primarily covered 94 candidate intervals across the entire *B. napus* genome (Table S3). Most repeated significant SNPs (1168/1431) were located in the interval qGSL-A09-5 with the most significant SNPs (S9_18653335) that explained 47.71% of phenotypic variance. In addition, the SNPs in the intervals qGSL-A01-4, qGSL-A06-2 and qGSL-C08-5 were repeatedly detected, which all explained more than 35% of the phenotypic variance (Table S1). Therefore, we believe that these regions are important interval regions for identifying the candidate genes for controlling the accumulation of m150. Furthermore, in total, 51 candidate regions are detected for m150, at least in one or any two years and BLUP values, suggesting that these remaining candidate regions are minor-effect intervals influencing the accumulation of m150 (Table S3). 

For the aliphatic glucosinolate m151, we identified 977, 629 and 747 significantly associated SNPs based on 2017cq, 2018cq and BLUP values, respectively, while only 225 SNPs were repeatedly detected in this study (Figure 3c and Figure S2c and Table S1). These significant SNPs primarily covered 67 candidate intervals across the *B. napus* genome, except for chromosome A01 (Table S3). Among the 225 SNPs, highly significant SNPs were repeatedly detected at intervals qGSL-A06-3, qGSL-A09-1, qGSL-A09-5 and qGSL-C07-6, which all explained more than 30% of phenotypic variance. Therefore, these regions should be considered as major interval regions controlling the accumulation of m151. In addition, 46 candidate regions with minor effects are detected for m151, at least in one or any two years and BLUP values (Table S3). 

For the aromatic glucosinolate m157, we identified 311, 550 and 1403 significantly associated SNPs based on 2017cq, 2018cq and BLUP values, respectively, 19 SNPs of which were repeatedly detected in this study (Figure 3d and Figure S2d and Table S1). These significant SNPs primarily covered 99 candidate intervals across the entire *B. napus* genome (Table S3). One significant association locus on chromosome C04 (named qGSL-C04-2) was detected, with the peak SNP S14_6056639, which explained 41.07% of phenotypic variance and was repeatedly detected in different environments (Table S3). Therefore, we used this interval region of chromosome C04 (5.85~6.27 Mb) to predict the candidate genes for controlling the accumulation of m157 in seeds. The interval region on chromosome A09 (named qGSL-A09-5) was also detected in different years, with the peak SNP S9_18294706, which explained 37.58% of phenotypic variance. Furthermore, S7_8312132 explaining 40.84% of phenotypic variance was identified and located on chromosome A07, named qGSL-A07-3. Thus, we believe that the interval regions (qGSL-A09-5, qGSL-A07-3 and qGSL-C04-2) are also closely related to the accumulation of m157 in rapeseed. Correspondingly, 79 candidate regions with minor effects were also detected for m157, at least in one or any two years and BLUP values (Table S3).

For the indole glucosinolate m165, we identified 50, 79 and 82 significantly associated SNPs based on 2017cq, 2018cq and BLUP values, respectively, containing 4 SNPs repeatedly detected in this study (Figure 3e and Figure S2e and Table S1). However, these significant SNPs primarily covered 22 candidate intervals located on chromosome A01, A02, A03, A05, A06, A07, A09, C01, C02, C04, C05, C08 and C09, respectively (Table S3). Three of these interval regions were repeatedly detected and located on chromosomes A06, A09 and C05, namely qGSL-A06-3, qGSL-A09-7 and qGSL-C05-3, respectively. The peak SNP mapped on qGSL-C05-3, qGSL-A06-3 and qGSL-A09-7 explained more than 20% of phenotypic variance (Table S3). In addition, the remaining intervals were detected for m165, at least in one or any two years and BLUP values (Table S3).

For the indole glucosinolate m172, we identified 211, 132 and 1035 significantly associated SNPs based on 2017cq, 2018cqs and BLUP values, respectively, including 47 repeatedly detected SNPs (Figure 3e and Figure S2f and Table S1). These significant SNPs primarily covered 50 candidate intervals across the *B. napus* genome, except the chromosome C09 (Table S3). For 47 SNPs, 6 significant SNPs were mapped on chromosome A01 (named qGSL-A01-4) with peak SNP S1_19207516, which explained 42.78% of phenotypic variance, and the peak SNP S9_18611734 was mapped on chromosome A09 (named qGSL-A09-5), explaining 38.62% of phenotypic variation. Therefore, these interval regions were used to predict the candidate genes for controlling the accumulation of m172 in seeds (Table S3). In addition, 43 candidate regions were detected for m172, at least in one or any two years and BLUP values (Table S3).

Altogether, we identified 113 candidate regions significantly associated with six glucosinolate metabolites, which were widely distributed throughout the *B. napus* genome. In particular, candidate interval regions qGSL-A07-3 and qGSL-A09-5 located on chromosome A07 and A09 were detected for five of the six glucosinolate metabolites (all except m165) in two years and BLUP values. In addition, qGSL-A01-4, qGSL-A02-2, qGSL-A03-3, qGSL-A05-2, qGSL-A06-1, qGSL-A06-3, qGSL-A09-6, qGSL-A09-7, qGSL-C04-2 and qGSL-C08-5 were repeatedly detected in at least one year or BLUP value. In these repeatedly detected interval regions, most of them were simultaneously associated with three glucosinolate metabolites (m145, m150 and m151) or any two of these (Table S3), perhaps because they are all aliphatic GSLs. Moreover, m145 is the precursor of m151 in the 4C pathway of aliphatic GSL biosynthesis in *B. napus*.

### 2.3. Candidate Gene Mining

Based on the physical positions of the significant SNPs, we mapped the intervals to the corresponding chromosomes of the reference genome *B. napus* Darmor-*bzh* (v4.1) [49] and searched for candidate genes for each significant locus based on the mGWAS data combined with the differentially expressed genes in BnHG vs. BnLG. In total, 187 candidate genes were identified and predicted for GSLs in this study, including genes encoding enzymes in the GSL biosynthesis pathway and their homologs, transcription factor genes and some novel candidate genes, respectively (Table S4). 

**Figure 4:**
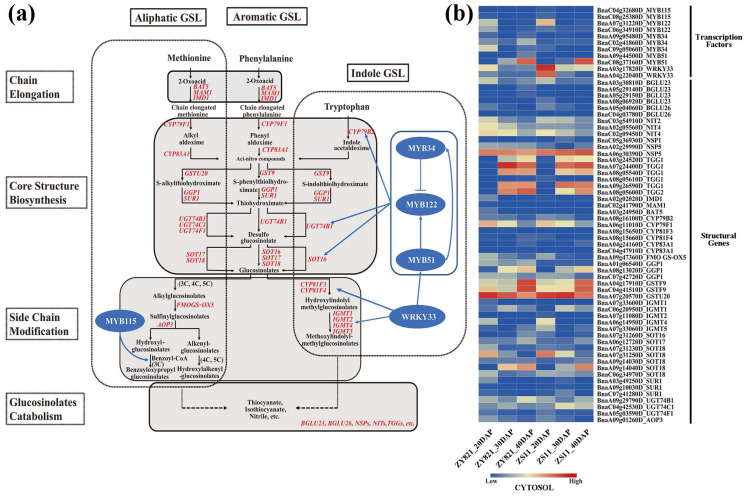
The proposed GSL biosynthesis pathway (**a**) and expression patterns of known candidate genes in *B. napus* (**b**). The proposed pathway was constructed based on published data [33,38,42,50,51,52,53]. BAT, bile acid transporter [54]; IMD, isopropylmalate dehydrogenase [55]; MAM1, methylthioalkylmalate synthase 1 [56]; CYP, cytochrome P450 [57]; UGT, UDP-glucosyl transferase [22,58]; GST, glutathione S-transferase [59,60]; GGP1, gamma-glutamyl peptidase 1 [61]; SUR1, S-alkyl-thiohydroximate lyase [31]; SOT, sulfotransferase [62]; FMOGS-OX, flavin-monooxygenase glucosinolate S-oxygenase [26]; IGMT, indole glucosinolate O-methyltransferase [63]; AOP3, 2-oxoglutarate-dependent dioxygenase [64]; BGLU, *β*-glucosidase [65]; NSP, nitrile specifier protein [66]; NIT, nitrilase [67]; TGG, thioglucoside glucohydrolase [68]. Genes with red color represent the identified candidate genes for six glucosinolate metabolites (Table S4). The expression levels of the candidate genes in BnLG and BnHG are listed in Table S5. Further, 20, 30 and 40 DAP represent the seeds after 20, 30 and 40 days after pollination, respectively.

**Figure 5:**
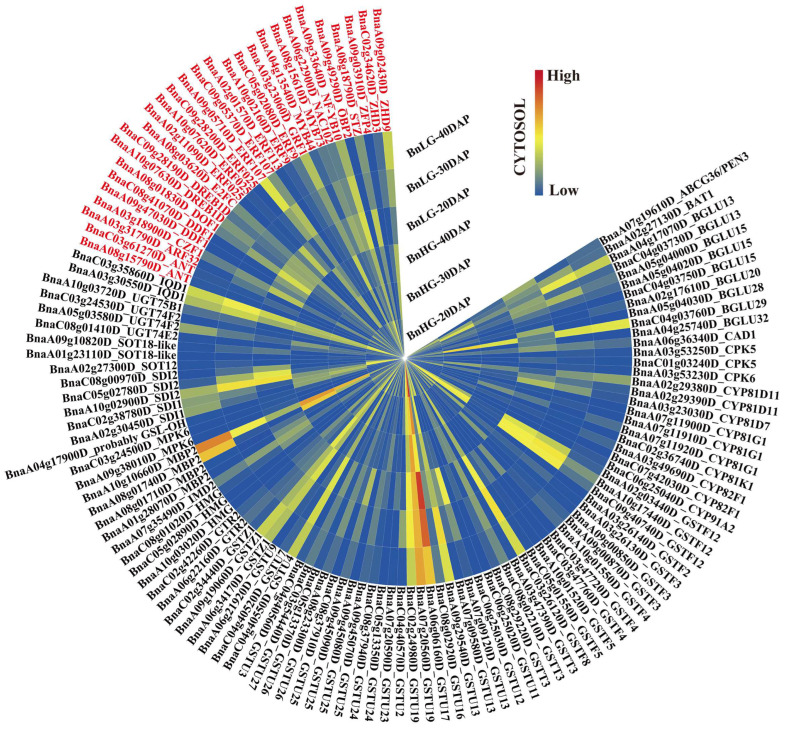
Expression patterns of the 123 novel candidate genes in *B. napus*. Genes shown in black are structural genes and those shown in red are transcription factor genes. The expression levels of these candidate genes in BnLG and BnHG are listed in Table S5. Further, 20, 30 and 40 DAP represent the seeds after 20, 30 and 40 days after pollination, respectively.

Based on the known GSL biosynthesis pathway, we identified 64 candidate genes in the GSL biosynthesis pathway (including genes encoding enzymes in this pathway and their homologs) among the intervals, which showed differential expression during seed development in BnHG vs. BnLG (Figure 4 and Figure 5, Table S4). 

Among these, key genes encoding enzymes that participate in the GSL biosynthesis pathway, such as genes encoding isopropyl malate dehydrogenase 1 (IMD1), methylthioalkyl malate synthase (MAM1), bile acid transporter (BAT5), cytochrome P450s (CYP79F1, CYP79B2, CYP83A1, CYP81F3 and CYP81F4), UDP-glucosyl transferases (UGT74B1, UGT74C1s and UGT74F1), glutathione S-transferases (GSTU20, GSTF9), *γ*-glutamyl peptidase 1 (GGP1), S-alkyl thiohydrogen oxidase lyase (SUR1), sulfotransferases (SOT16, SOT17 and SOT18), flavin-monooxygenase glucosinolate S-oxygenase 5 (FMOGS-OX5), oxoglutarate-dependent dioxygenase (AOP3) and indole-glucoside O-methyltransferases (IGMT1, IGMT2, IGMT4 and IGMT5), were identified on the following chromosomes: A01 (*GGP1*, *BnaA01g06540D*), A02 (*IMD1*, *BnaA02g02020D*), A03 (*SUR1*, *BnaA03g49250D;* and *BAT5*, *BnaA03g24950D*), A04 (*CYP83A1*, *BnaA04g24160D;* and *GSTF9*, *BnaA04g17910D*), A05 (*UGT74F1*, *BnaA05g03590D*), A06 (*CYP79F1*, *BnaA06g11010D*; *IGMT4*, *BnaA06g14950D;* and *SOT17*, *BnaA06g12720D*), A07 (*GSTU20*, *BnaA07g20570D*; *IGMT1*, *BnaA07g33600D*; *IGMT2*, *BnaA07g11080D*; *IGMT5*, *BnaA07g33060D*; *SOT16*, *BnaA07g31260D*; *SOT18*, *BnaA07g31230D;* and *BnaA07g31250D*), A08 (*CYP79B2*, *BnaA08g16100D*; *CYP81F3*, *BnaA08g15650D*; *CYP81F4*, *BnaA08g15660D;* and *GGP1*, *BnaA08g13020D*), A09 (*FMOGS-OX*, *BnaA09g47360D*; *SOT18*, *BnaA09g14030D;* and *BnaA09g14040D*; *SUR1*, *BnaA09g10030D*; *UGT74B1*, *BnaA09g29790D*; and *AOP3*, *BnaA09g01260D*), *C02* (*MAM1*, *BnaC02g41790D*), C04 (*CYP83A1*, *BnaC04g47910D*; *GSTF9*, *BnaC04g41510D;* and *UGT74C1*, *BnaC04g42530D*), C06 (*IGMT1*, *BnaC06g20950D;* and *SOT18*, *BnaC06g34970D*) and C07 (*GGP1*, *BnaC07g42720D;* and *SUR1*, *BnaC07g41280D*), respectively (Figure 4, Table S4), indicating that our GWAS approach was successful in this study.

Previously studies have identified MYB transcription factors that regulate GSL biosynthesis in various plants [36,38,50,69]. In the current study, we identified 39 transcription factors that were predicted to regulate GSL biosynthesis, including 15 AP2/ERF transcription factors (ERFs, ARF3, DREBs and ANT), seven zinc finger proteins (CZF1, STZ, ZHDs, DOF1, OBP2 and ZFP4), two WRKY transcription factors (WRKY33), one growth regulator (GRF1), one NAC family protein (NAC102), one E2F transcription factor (E2FC), one nuclear transcription factor Y subunit B (NF-YB10) and 11 MYB transcription factors (MYBs). Among these, homologous genes of *MYB34*, *MYB51*, *MYB122* and *WRKY33*, which are involved in GSL biosynthesis [42,70], were identified on the interval regions of chromosomes A03, A04, A07, A09, C02, C06, C08 and C09, respectively (Table S4). We also identified some novel transcription factor genes (e.g., MYB44, ERF025, NF-YB10 and E2FC) associated with the significant SNPs, with obvious differences in expression in developing seeds of BnHG and BnLG (Figure 5, Table S4). These transcription factors might play an important role in the biosynthesis of GSLs in *B. napus*.

In addition, we identified 29 candidate genes encoding enzymes responsible for the hydrolysis of GSLs, including genes encoding three nitrile hydrases (NITs), three nitrile specifier proteins (NSPs), one glutathione gamma-glutamylcysteine transferase (PCS1), fifteen *β*-glucosidases (BGLUs), one ABC transporter family protein (PEN3) and six glucosidolate glucohydrolases (TGGs), respectively (Figure 4 and Figure 5, Table S4). Correspondingly, we also identified two calmodulin-binding proteins (IQD1), three structural genes (HMGs), four genes encoding myrosinase-binding proteins (MBP2) and five protein kinase genes (CPKs and MPK6) located in the interval regions for indole GSLs (Figure 5, Table S4). Among these, WRKY33, CPK5 and MPK6 were shown to mediate indole GSL biosynthesis [71], which in consistent with our results. We also identified two glucosinolate transporters (GTR2) and five TRP family proteins (SDIs) (Figure 5, Table S4).

Altogether, numerous key homologous genes, including known genes (such as *IMD1*, *MAM1*, *IGMTs*, *MYB34*, *MYB51*, *MYB122* and so on) and novel genes (*MYB44*, *ERF025*, *ARF3*, *NF-YB10*, *E2FC* and so on) were identified within the confidence intervals of GSLs. Our findings not only demonstrate the reliability of the association genetics approach, but also provide new insight into elucidating the biosynthesis of these GSLs in *B. napus* seeds.

## 3. Discussion

GSLs are well-known secondary metabolites that play important roles in plant defense against diseases and insects and in human nutrition/health [1,2,3,72]. However, some glucosinolates in seed meal have deleterious effects on poultry and livestock, leading to efforts to develop low-glucosinolate *Brassica* crops [43,73]. Based on the functional group of amino acids, GSLs are divided into three major types, namely aliphatic GSLs, indole GSLs and aromatic GSLs, respectively [12,13,14]. Aliphatic GSLs comprise a major proportion of total GSLs in seeds [74,75,76]. In this study, we identified six major GSL metabolites in rapeseed, including three aliphatic GSLs (m145 gluconapin, m150 glucobrassicanapin and m151 progoitrin), one aromatic GSL (m157 gluconasturtiin) and two indole GSLs (m165 Indolylmethyl glucosinolate and m172 4-hydroxyglucobrassicin) (Figure 1 and Figure 2, Table 1), respectively. However, among the six GSLs examined, four showed highly significant differences in abundance in BnHG vs. BnLG seeds, whereas two indole GSLs did not (Figure 2a). It seems that aliphatic GSLs account for most of the total GSL content, comprising an average of 1539.35~1975.56 μg/g FW in both years of the study. We detected a higher positive correlation between the contents of aliphatic GSLs and aromatic GSLs vs. indole GSLs (Figure 1c), supporting the notion that aliphatic GSLs and aromatic GSLs are somewhat correlated in *B. napus* seeds. However, deeper knowledge of the specific GSLs in *B. napus* seeds is required to help breeders improve the current varieties and select plants with advantageous properties.

Unlike in the model plant *Arabidopsis thaliana*, many copies of homologous genes involved in the GSL biosynthesis pathway are present in the rapeseed genome, as *B. napus* is an allopolyploid plant with a complex genome [49]. However, numerous studies, including quantitative trait locus (QTL) mapping and candidate gene identification, have investigated the mechanisms involved in GSL production in rapeseed. For example, numerous QTL for GSLs in leaves and seeds of *B. napus* have been detected [77,78]. For seed GSL content, Xu et al. [79] and Wang et al. [80] also identified the significantly associated sites located on chromosomes A9, C2 and C9, respectively. We previously identified 11 significant SNPs associated with seed GSL accumulation in *B. napus* located on chromosomes A08, A09, C03 and C09 [81]. Tan et al. [82] recently detected 15 reliable quantitative trait loci (QTLs) for seed GSL content via a GWAS. With the rapid development of gene chip and genome sequencing technology, mGWAS combined with genomic and transcriptomic analysis is suitable for studying the metabolism, genetic characteristics and biochemical properties of plants, and has been widely used for structural and functional analysis of metabolites and functional genomics [83,84,85]. In the current study, we detected 113 candidate intervals with significant associations with glucosinolate metabolites in rapeseed via mGWAS (Figure 3, Table S3). Of these, 13 candidate intervals were significantly associated with only a single metabolite, while 100 were associated with multiple metabolites. Importantly, we repeatedly detected the significant interval regions on chromosomes A02, A08, A09, C03, C07 and C09, which is consistent with previous studies [77,78,81,82,86,87,88,89]. We also identified many new candidate intervals located on chromosomes A04, C01, C05, C06 and C08, further demonstrating the power of our approach (Figure 2, Table S3).

Producing seeds with little or no GSL represents an important breeding objective of *B. napus* in the past few decades. Indeed, ideal rapeseed has been developed containing high GSL levels in vegetative tissues and little or no GSL in mature seeds by allele mining [78,90]. Numerous studies have also revealed many key genes involved in GSL biosynthesis in *B*. *napus*, such as *GTR2* [82], *MYB28* [91], *MAM1*, *CYP83A1, UGT74B1* [92] and *LEC1* [93]. In this study, we identified two candidate genes involved in amino acid side chain extension, *BnaMAM1* (*BnaC02g41790D*) and *BnaIMD1* (*BnaA02g02020D*), which are located in candidate intervals qGSL-A02-1 and qGSL-C02-11, associated with three aliphogenic GSLs and one aromatic GSL (Table S4), and showed higher expression profiles in BnHG vs. BnLG (Figure 4, Table S5). Furthermore, their homologous genes in *A. thaliana* are involved in the biosynthesis of GSLs [56,94]. We also identified *BnaFOMGS-OX5* (*BnaA09g47360D*) and *BanGS-OH* (*BnaA04g17900D*), which participate in the side chain modification process of aliphatic GSL biosynthesis and showed higher expression profiles in BnHG vs. BnLG (Figure 4 and Figure 5, Tables S4 and S5) [95,96]. In addition, several genes encode major enzymes that catalyze the production of the core structures of GSL, including *CYP79* genes [97], *CYP83* genes [20], *GGP1* [43], *SUR1* [31], *UGT74* genes [22] and *SOT* genes [62]. We identified homologs of these genes, such as *BnaCYP79F1* (*BnaA06g11010D*), *BnaCYP79B2* (*BnaA08g16100D*), *BnaCYP83A1* (*BnaA04g24160D* and *BnaC04g47910D*), *BnaGGP1* (*BnaA01g06540D*, *BnaA08g13020D* and *BnaC07g42720D*), *BnaSUR1* (*BnaA09g10030D*), *BnaUGT74B1* (*BnaA09g29790D*), *BnaUGT74C1* (*BnaC04g42530D*), *BnaSOT16* (*BnaA07g31260D*), *BnaSOT17* (*BnaA06g12720D*) and *BnaSOT18* (*BnaA07g31230D*, *BnaA07g31250D*, *BnaA09g14030D*, *BnaA09g14040D* and *BnaC06g34970D*) (Figure 4, Table S4 and S5). In addition, understanding GSL metabolites is important for targeting genes in the relevant biosynthetic pathways. Here, we identified the homologs of genes involved in diverse aspects of GSL biosynthesis and accumulation in *B. napus*, such as *BGLU* genes (15), *NSP* genes (3), *CAD1* (1), *TGG* genes (6) and *NIT* genes (3) (Figure 4, Table S4 and S5), which are essential for the catabolism and hydrolysis of GSL metabolites [66,67,69,98,99]. Importantly, some novel genes in the GSL biosynthesis pathway were also identified. For example, *BnaSOT12* (*BnaA02g27300D*) is homologous to *AtSOT12*, which functions in flavonoid, brassinosteroid and salicylic acid activity, and is involved in plant responses to salt, osmotic stress and phytohormones [100,101]. The candidate genes, *BnCYP81D11* (*BnaA02g29380D* and *BnaA02g29390D*), *BnaCYP81D7* (*BnaA03g23030D*), *BnaCYP81G1* (*BnaA07g11900D*, *BnaA07g11910D* and *BnaA07g11920D*), *BnaCYP81F3* (*BnaA08g15650D*), *BnaCYP81F4* (*BnaA08g15660D*) and *BnaCYP81K1* (*BnaC02g36740D*), are located in the interval regions associated with glucosinolate metabolites and showed differential expression in BnHG vs. BnLG seeds (Figure 4 and Figure 5, Tables S4 and S5). Among them, *CYP81F1* and *CYP81F3* are known to be involved in indole GSL biosynthesis [102,103], but there are few reports of these genes in *B. napus*. In addition, 46 related transferase genes, including *GSTFs*, *GSTU* and *GSTT* genes along with *GSTZ*, showed different expression levels in BnHG vs. BnLG seeds (Figure 5, Tables S4 and S5). Similarly, some of these genes are known to be involved in GSL biosynthesis in other species [1,104].

GSLs play diverse roles in plant defense, and several transcription factors have been identified as important regulators of GSL biosynthesis. For example, WRKY33 directly regulates indolic glucosinolate (IGS) biosynthesis, specifically the production of 4-methoxyindole-3-ylmethyl glucosinolate (4MI3G), by directly activating the expression of *CYP81F2*, *IGMT1* and *IGMT2* [42], while the R2R3-MYB transcription factors, MYB51, MYB34, MYB122 and MYB115, regulate aliphatic and indole GSL biosynthesis [35,50,69,70,105]. In addition, the zinc finger protein OBP2 has been implicated in indole GSL biosynthesis in *A. thaliana* [106]. In this study, we identified genes encoding various transcription factors, such as MYBs (MYB34, MYB51, MYB122 and MYB115), AP2/ERFs (ERF9), WRKY (WRKY33) and Zinc finger protein (OBP2), located in the interval regions for GSL metabolites (Figure 4 and Figure 5, Tables S4 and S5). In addition, we identified some new transcription factors, including MYB44 (BnaA04g13540D), DREB1D (BnaA10g07630D and BnaC09g28190D), NAC102 (BnaA06g22900D), NF-YB10 (BnaA09g33640D) and E2FC (BnaA08g03620D), that showed differential gene expression profiles in BnHG vs. BnLG, suggesting they might be involved in regulating GSL biosynthesis in *B. napus* (Figure 5, Tables S4 and S5). Our findings suggest that the different candidate homologous genes play conserved roles in GSL biosynthesis, although their biological functions in *B. napus* must be confirmed through careful analysis. 

In conclusion, we identified 113 interval regions for six glucosinolate metabolites in *B. napus* seeds (Figure 3, Table S3). We also identified 187 candidate genes for GSL biosynthesis and accumulation. Our results enrich our knowledge of the GSL biosynthesis pathway and provide candidate genes for improving the compositions of specific GSLs in rapeseed, which could be precisely modified by gene editing in the future.

## 4. Materials and Methods

### 4.1. Plant Materials

In total, 143 *B*. *napus* accessions were collected from major breeding institutes across China, including 70 low-glucosinolate content (<45 μmol/g), 46 medium-glucosinolate content (45~100 μmol/g) and 27 high-glucosinolate content (>100 μmol/g), respectively (Table S6). All accessions were grown in the growing seasons of 2017–2018 and 2018–2019 (namely 2017Cq and 2018Cq, respectively) in Chongqing, China. The field experiments are designed as in our previous research [107]. In brief, all field experiments were carried out by a randomized complete block design, with three biological replications across each environment. Each accession was grown in a plot with three rows, singling 10–12 plants each row. At the stage of flower initiation, the individual flowers were marked on each plant to ensure that seeds are at the same stage of development. The fresh seeds at 35 days after flowering (DAF) were sampled and pooled from five or more individuals, and quickly frozen in liquid nitrogen. The representative *B*. *napus* accessions with high (Zhongyou 821, BnHG) and low (Zhongshuang 11, BnLG) seed glucosinolate content were selected from 143 *B*. *napus* accessions for transcriptome sequencing (RNA-Seq), respectively. The seeds of 20, 30 and 40 DAF were also collected and pooled from five or more individuals in BnHG and BnLG plants. All samples were stored at −80 °C until further analysis.

The total RNA was extracted from the seeds of 20, 30 and 40 DAF using an EZ-10 DNAaway RNA Mini-Preps kit (Sangon Biotech, Shanghai, China) following the manufacturer instructions. Then, the qualified RNA samples were used for libraries construction and sequenced on Illumina Hiseq 2000 platform with 150 bp paired-end reads (Tianjin Novogene Bioinformatic Technology Co., Ltd., Tianjin, China). The gene expression profiles were evaluated using FPKM (fragments per kilo base of exon model per million) values. Genes were considered as differentially expressed genes (DEGs) with a minimum 2-fold difference in expression (|log2FC| ≥ 1). 

### 4.2. Glucosinolate Matbolite Extraction

The raw metabolites were extracted from fresh seeds described in our previous research [52], with minor modifications. In short, about 100 mg of fresh seeds was crushed into powder using high-throughput tissue grinder (Tissuelyser-192, Shanghai, China). Subsequently, we added the 500 μL extract solution (80% aqueous methanol with 0.1% formic acid) and homogenized by vortex for 10 s. The homogenized extraction buffer was extracted using sonication (KQ-100E, Kunshan, China) at 4 °C for 1 h, followed by centrifugation at 12,000× *g* at 4 °C for 10 min. Then, the residues were repeatedly extracted. Eventually, the mixed liquid supernatants were used for UPLC-HESI-MS/MS analysis after being filtered by a 0.22 μm nylon filter. All experiments were performed in at least three replicates for each accession.

### 4.3. UPLC-HESI-MS/MS Analysis

The UPLC-HESI-MS/MS was performed using Dionex UltiMateTM 3000 UHPLC system (Thermo Fisher Scientific, Waltham, MA, USA) coupled to a Thermo Scientifific Q-Exactive System equipped with an S-Lens ionizer source (Thermo Scientifific, Waltham, MA, USA), including the precolumn (pore size: 1.7 μm, 2.1 × 5 mm, Waters, Wexford, Ireland) and Acquity UPLCBEH C18 column (pore size: 1.7 μ m, 2.1 mm × 150mm, Waters, Wexford, Ireland). The parameters were as follows: the mobile phases A (0.1% formic acid) and B (0.1% acetonitrile aqueous solution), 37 °C column temperature, 0.3 mL/min flow rate and 10 μL injection volume, respectively. The mobile phase gradients are 0–2 min, 5% B–10% B; 2–10 min, 10–25% B; 10–13 min, 25–95% B; 13–16 min, 95% B; 16–16.5 min, 95–5% B; 16.5–21 min, 5% B. The mass spectrometry was detected in negative ion mode with a scanning range of 100 to 1200 (m/z), 3.5 kV ion source voltage, 350 °C capillary temperature, 35 sheath gas, 10 auxiliary gas and 0 backblow air, respectively.

### 4.4. Data Processing and Glucosinolate Metabolite Identification

Raw data of UPLC-HESI-MS/MS were firstly treated with MS-DAIL ver4.1 MSP negative database (http://prime.psc.riken.jp/compms/msdial/main.htmL#MSP, accessed on 2 April 2022), and automatically converted by ABF (Analysis Base File) converter (http://www.reifycs.com/AbfConverter/index.html, accessed on 12 March 2020) [108]. Correspondingly, the glucosinolate metabolites were identified using Xcalibur ver4.1 based on retention time (RT), MS and MS/MS spectral data, referring to the published information [109,110]. Furthermore, the amounts of six glucosinolate metabolites were quantified by drawing standard curve of sinigrin [110,111], and the calibration curve was constructed using eight points generated from the sinigrin concentration gradients, ranging from 0.001 to 2 mg L^−1^ (0.001, 0.005, 0.01, 0.05, 0.20, 0.50, 1.0 and 2.0 mg L^−1^). 

The glucosinolate metabolites were detected in two consecutive years (2017 and 2018) with replications; we further obtained the best linear unbiased prediction (BLUP) of six glucosinolate metabolites per accession using a linear model using an R script (http://www.eXtension.org/pages/61006, accessed on 14 October 2020), respectively. The content of six glucosinolate metabolites in the 2017 and 2018 growing seasons and resulting values of BLUP were used as phenotypes for GWAS, respectively. The heritability was calculated by using the multi-year repeated model. In addition, the quantitative data of glucosinolate metabolites were divided into 10 grades, and Shannon–Wiener Diversity Index was used to calculate metabolites [112,113,114]. The Pearson correlation coefficients among six glucosinolate metabolites were calculated by the R language psych package and significance tests (Student *t*-test) were performed [115]. Statistical analysis of glucosinolate metabolites was performed using Microsoft Office Excel 2009. The G × E analysis of glucosinolate metabolites was performed using AMMI model by R package “agricolae”.

### 4.5. GWAS of Glucosinolate Metabolites

Detailed methods used for SNP genotyping and mapping were previously described [46,47,48]. In brief, DNA libraries with a mean insert size of 350 bp were constructed, and 125 bp paired-end reads were generated using an Illumina HiSeq 4000 instrument at the Biomarker Technologies Corporation (Beijing, China). Low-quality bases from paired-end reads were trimmed using Trimmomatic (version 0.33) and mapped to the rapeseed genome ‘Darmor*-bzh*’ [49] using the Burrows–Wheeler Aligner (version 0.7.10-r789). Then, we used Picard (release 2.0.1, http://broadinstitute.github.io/picard/, accessed on 26 July 2018) and GATK (version 3.2) to process local realignment and base quality detection for the alignment results, sequentially. Further, we then used AMtools mpileup (version 0.1.19–44428cd) and GATK to perform SNP calling. In total, 239,945 high-quality SNPs with a minor allele frequency (MAF) <5% were used for further analysis. The six models, including naïve, Q, K, PCA, K + Q and K + PCA model, were applied to determine the statistical associations between phenotypes and genotypes. Quantile–quantile (QQ) plots were used for false-positive correction for association analyses. In this study, genome-wide association analysis for six glucosinolate metabolites was carried out using the GLM with Q model and MLM with Q + K model by TASSEL 5.2.1 software. The population structure Q matrix was completed by admixture_linux-1.3.0 software [116]. The significant signal of the associations between SNPs and the glucosinolate metabolites was assessed based on the threshold *P*  <  *P*  =  1/N (where N is 239,945 SNPs in this study), and the threshold of significance was set to *p* < 4.17 × 10^−6^.

### 4.6. Annotation of Candidate Genes

The significant interval regions were mapped to the Darmor-*bzh* reference genome (version 4.1, http://www.genoscope.cns.fr/brassicanapus/data/, accessed on 26 October 2022) [49], which were anchored by the physical position of significant SNPs. Correspondingly, 200 kb flanking region of association SNPs was used as the candidate interval region. Then, the annotated genes in the interval regions were used for screening the candidate genes, and further confirmed by gene expression analysis. Herein, the gene expression levels were calculated as FPKM (Fragments Per Kilobase of transcript sequence per Millions base pairs) with the featureCounts tool in Subread [117]. Finally, correlations between the GSL phenotype and the gene expression profiles were detected to determine the candidate genes that were associated with the glucosinolate metabolites.

## Figures and Tables

**Table 1:** Phenotypic variations for six glucosinolate metabolite contents in an association panel of *B. napus*.

Name	No. ^1^	Env. ^2^	Min. ^3^	Max. ^4^	Avg. ^5^	SD ^6^	CV ^7^	Skew. ^8^	Kurt. ^9^	H’ ^10^	*F_ge_* ^11^	Her. ^12^
aliphatic GSLs	m145	2017cq	0.00	3007.14	456.59	584.04	1.28	1.59	2.33	1.37	57.46 **	0.63
		2018cq	0.00	943.99	125.82	222.02	1.76	2.21	4.12	1.07		
		mean	0.00	1975.56	291.20	403.03	1.52	1.90	3.23	1.22		
	m150	2017cq	0.00	2538.57	278.17	473.62	1.70	2.41	6.06	1.09	59.12 **	0.80
		2018cq	0.00	1407.20	146.98	276.58	1.88	2.56	6.58	1.04		
		mean	0.00	1972.88	212.58	375.10	1.79	2.48	6.32	1.06		
	m151	2017cq	0.01	2418.94	374.82	534.24	1.43	1.63	1.94	1.20	86.47 **	0.52
		2018cq	0.00	659.77	70.16	138.30	1.97	2.48	5.68	0.97		
		mean	0.00	1539.35	222.49	336.27	1.70	2.06	3.81	1.08		
aromatic GSL	m157	2017cq	0.00	594.24	59.27	108.99	1.84	2.95	9.57	0.87	87.95 **	0.49
		2018cq	0.00	152.39	18.24	31.70	1.74	2.32	5.06	1.06		
		mean	0.00	373.31	38.75	70.35	1.79	2.64	7.31	0.96		
indole GSLs	m165	2017cq	0.00	1071.04	435.89	231.28	0.53	0.57	0.23	2.09	18.36 **	0.66
		2018cq	1.24	763.78	248.89	183.86	0.74	0.54	−0.33	1.88		
		mean	0.62	917.41	342.39	207.57	0.63	0.56	−0.05	1.98		
	m172	2017cq	0.00	840.19	357.75	152.00	0.42	0.20	0.10	1.97	17.62 **	0.42
		2018cq	8.66	370.66	159.13	74.23	0.47	0.31	0.11	1.90		
		mean	4.33	605.42	258.44	113.12	0.45	0.26	0.10	1.93		

cq, Chongqing environment; ^1^ No., metabolite ID; ^2^ Env., environment; ^3^ Min., minimum; ^4^ Max., maximum; ^5^ Avg., average; ^6^ SD, standard deviation; ^7^ CV, coefficient of variation; ^8^ Skew., Skewness; ^9^ Kurt., Kurtosis; ^10^ H’, Shannon–Wiener diversity index; ^11^ *F_ge_*, the *F*-values for G × E for glucosinolate content. Asterisks indicate significant differences (**, *p* < 0.01). ^12^ Her., heritability. Metabolite content is expressed in μg/g FW.

## Data Availability

All other datasets supporting the results of this article are included within the article and Supplementary Materials.

## Supplementary Materials

The following supporting information can be downloaded at: https://www.mdpi.com/article/10.3390/plants12030639/s1, Figure S1: The QQ plots of 6 glucosinolate metabolites; Figure S2: Manhattan plots of association analysis for six glucosinolates using Q+K model; Figure S3: The significant SNPs associated with 6 glucosinolates in different environments; Figure S4: Correlation analysis of six glucosinolate metabolites in 2017cq and 2018cq; Table S1: Summary of repeatedly detected SNPs for 6 glucosinolate metabolites by mGWAS; Table S2: Summary of interval regions for 6 glucosinolate metabolites in *B. napus*; Table S3: Candidate interval regions for 6 glucosinolate metabolites in *B. napus*; Table S4: Candidate genes for 6 glucosinolate metabolites in *B. napus*; Table S5: The expression levels of the candidate genes in BnHG and BnLG of *B. napus*; Table S6: The list of 143 *B*. *napus* accessions used in this study; Table S7: Statistical analysis of 6 glucosinolate metabolites in high-, medium- and low-glucosinolate-content rapeseed
