# Supplementary figures and images for: Genome-Wide Association Study of Glucosinolate Metabolites (mGWAS) in Brassica napus L
Source: Plants (Basel). 2023 Feb 1;12(3):639. doi: 10.3390/plants12030639 (PMC9921834; doi:10.3390/plants12030639)

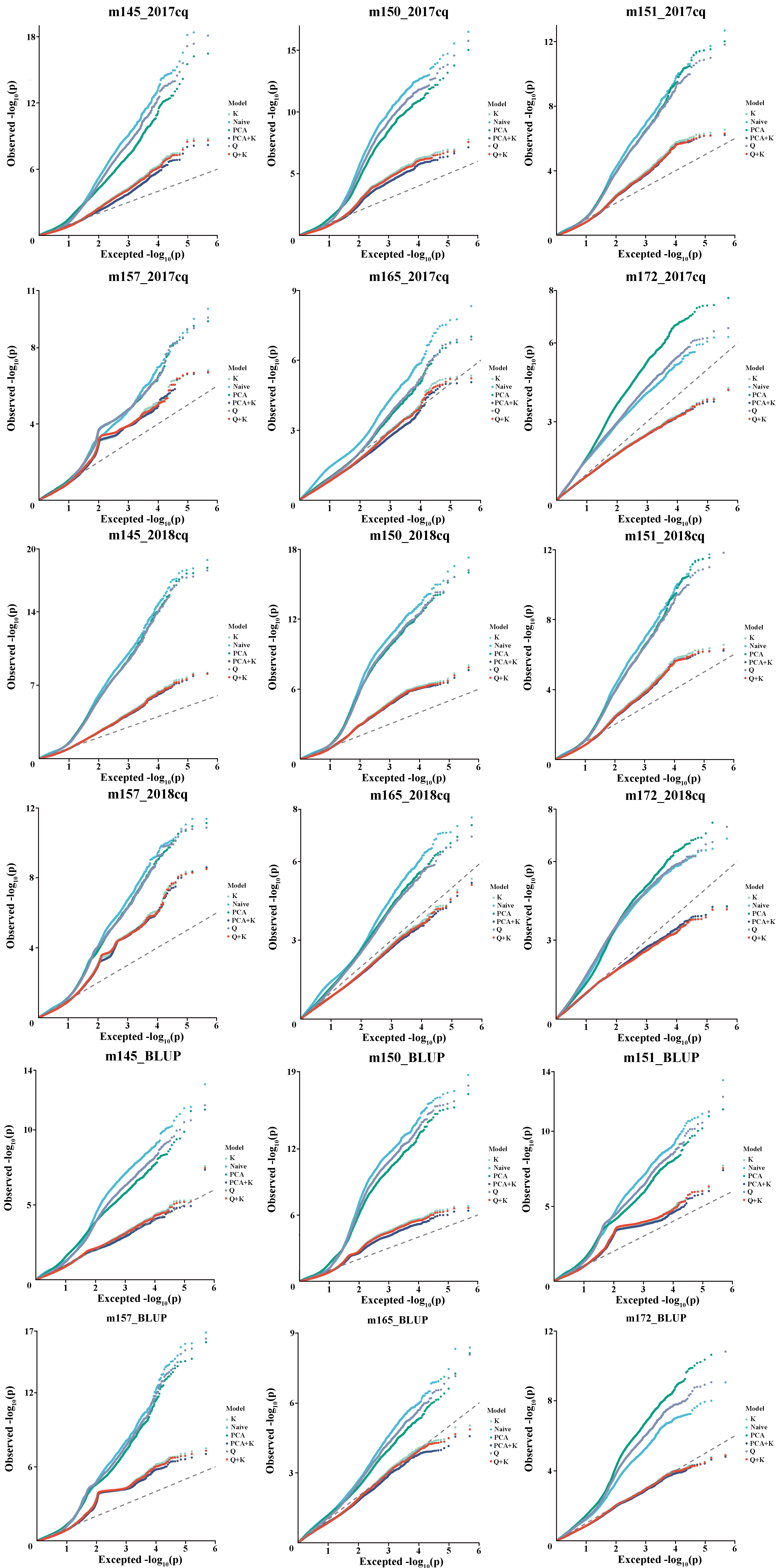

Supplement: Supplementary file 1 [file plants-12-00639-s001.zip › Figure S1. The QQ-plots of 6 glucosinolate metabolites.jpg]

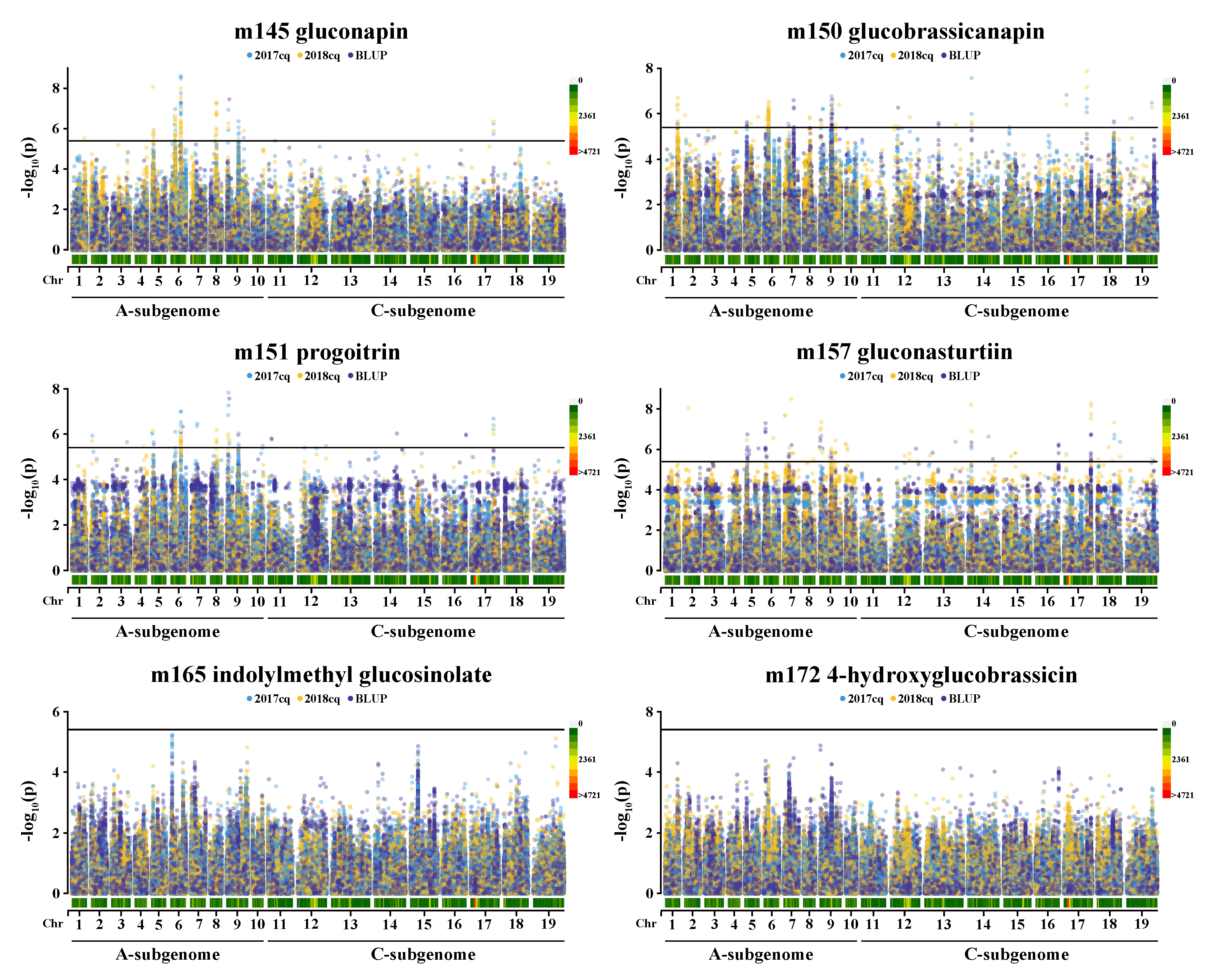

Supplement: Supplementary file 1 [file plants-12-00639-s001.zip › Figure S2. Manhattan plots of association analysis for six glucosinolates using Q+K model.jpg]

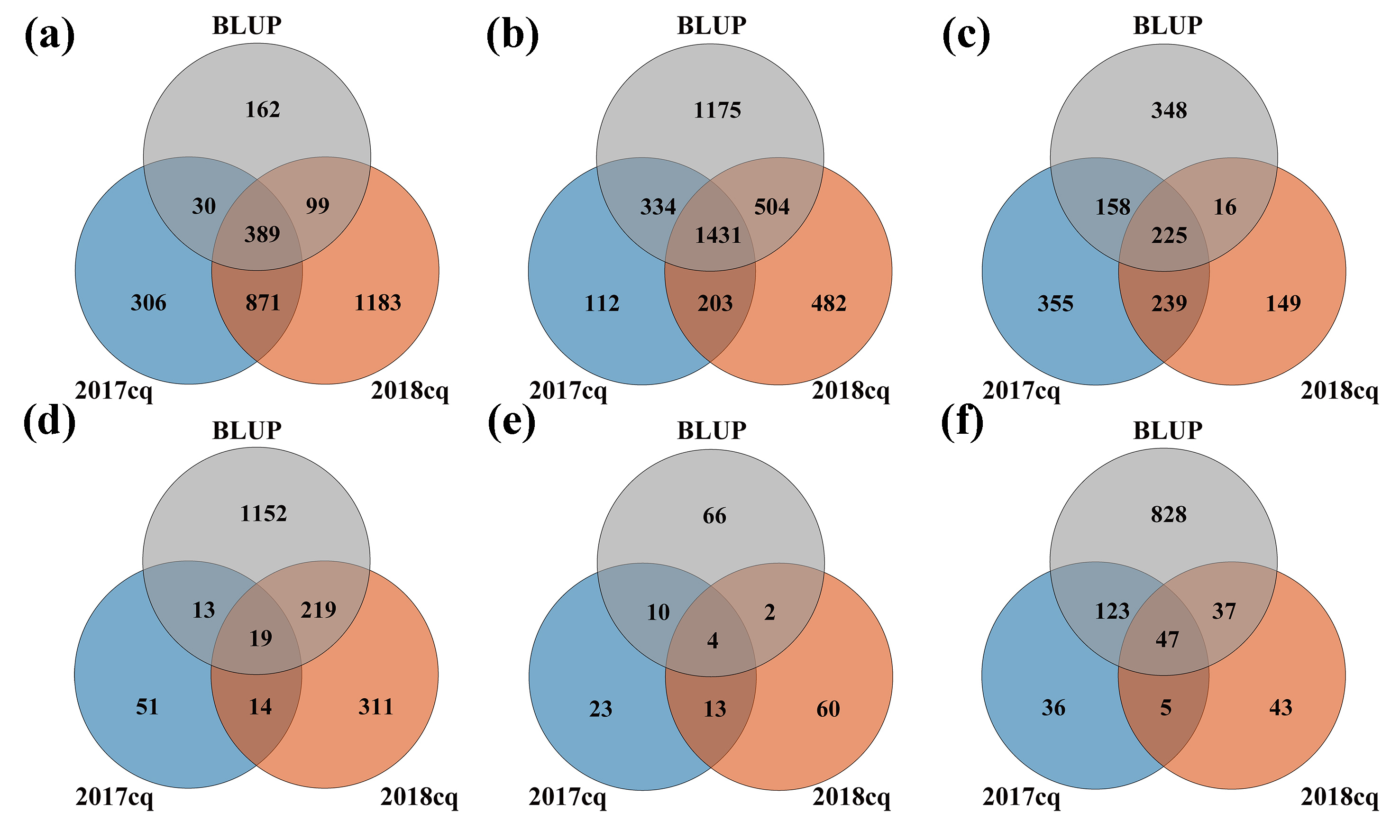

Supplement: Supplementary file 1 [file plants-12-00639-s001.zip › Figure S3. The significant SNPs associated with 6 glucosinolates in different environments.jpg]

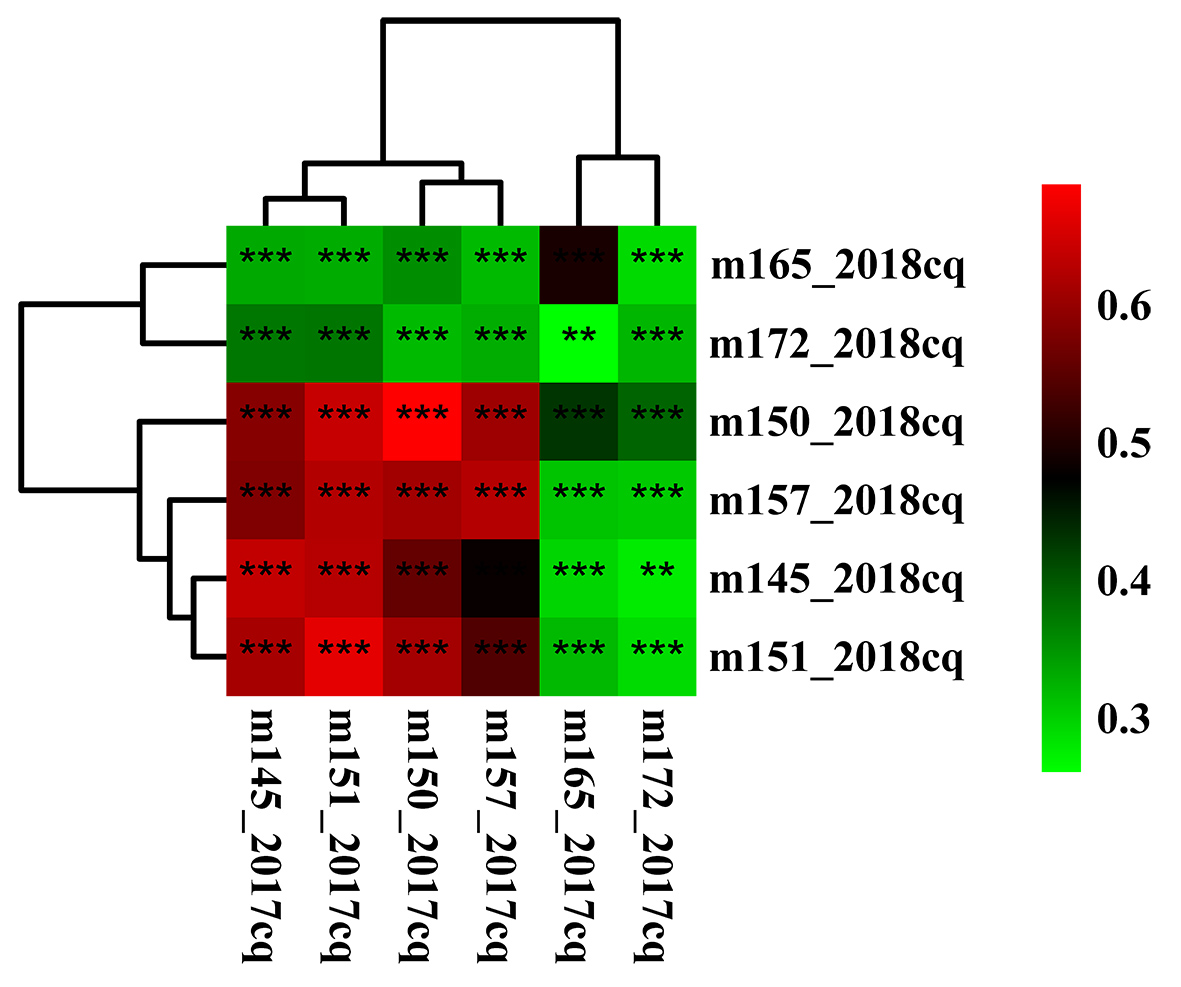

Supplement: Supplementary file 1 [file plants-12-00639-s001.zip › Figure S4. Correlation analysis of six glucosinolate metabolites in 2017cq and 2018cq.jpg]
